# R.B.N.A. Speakers' Hostility to the College: Lay Governors and Hospital Chairmen Attacked

**Published:** 1918-02-16

**Authors:** 


Chairman of the Council of the College of Nursing.

P.S.—To avoid possible misconception on the part of the public, I ought perhaps to add that the College of Nursing stands, as it has from the first, for the State Registration of Nurses, for a uniform curriculum, for a one-portal examination, and in the case of Nurses admitted to its Register after the expiration of the period of grace for not less than three years' training in a recognised hospital or infirmary.

### STATE REGISTRATION.

### R.B.N.A. Speakers' Hostility to the College.

### LAY GOVERNORS AND HOSPITAL CHAIRMEN ATTACKED.

### Liverpool Speakers Favour Uniting Forces.

A meeting promoted by the Royal British Nurses' Association was held in the Royal Institution, Liverpool, on Friday, February 8, 1918.

Nurses in the Homes of the People.

Dr. E. W. Hope (Medical Officer of Health for Liverpool), who presided over a large audience, said everything which could be done to advance either the efficiency of nursing or the welfare of nurses would always meet with very warm acceptance in Liverpool. His own personal experience of the valuable work done by nurses extended over something like thirty-five years. At a very early period in his official life he was greatly impressed by their work done, not only in the hospitals, but also in the homes of the people. He had no intention of putting the two into comparison; it would be a futile thing to do. Both branches of nursing were so extremely important that one could not take precedence over the other. Of late years the terrible events which they had been passing through, although they had increased the demands upon the nursing profession in the hospitals, had been not less insistent upon the services of the nurses in the homes of the people. In Liverpool alone more than 3,000 hospital beds had been handed over for the wounded and sick soldiers from overseas. That meant that nurses must be provided for them, and it also meant that those 3,000 beds, which were occupied by infirm people, the sick, and the wives and families of the soldiers, must be turned out to make room for the wounded soldiers. They had to go to their own homes. It was largely to the nursing profession that they looked, and they did not look in vain, to supply, as far as possible, every comfort to which those sick people had been accustomed. That was one of the debts they owed to the nursing profession, and one which he feared would be very little realised, but it was none the less an extremely important one. There was yet another great service which the nursing profession had been rendering to the community. There had not been a sufficient accommodation for the infectious sick; consequently, the Health Committee of Liverpool, and no doubt similar bodies in other centres, had added to their staffs of outdoor nursing—nurses qualified and trained for that specific work also. He had mentioned those things to refresh their memories as to the lines upon which the nursing profession was becoming more and more valuable, and more and more entitled to their gratitude and support and respect.

Miss Macdonald (Secretary to the Royal British Nurses' Association) after denouncing at considerable length the action of the British Women's Hospital Committee as an appeal for charity and her profession pauperised, said: They would ask her what advantages membership of the Royal British Nurses' Association offered. First of all, it was a corporate body, which could move and act as one body. It could defend itself and its members in a Court of Law. It could take over and administer funds for the benefit of nurses. It could undertake financial responsibility without its members being liable. It had inaugurated various schemes from time to time for the benefit of its members. There were four private nursing staffs, three in London and one in Australia, which were established on co-operative principles for the benefit of the nurses. The nurses received their fees from the patients, less a small percentage for working expenses. Then, although the Association was not in any way to be regarded as an employment bureau, they had found a considerable number of permanent appointments for their members. In that they very often had the co-operation of their members to a very great extent. The Association conferred a diploma in nursing upon such fully qualified nurses as passed a higher examination in nursing. Their Association was the only body which granted a diploma in nursing, and it did that under powers conferred by its Royal Charter. There was a nurses' library at their headquarters in Orchard Street, and a library of general literature at the Settlement Home in Clapton Square. That Home was provided and maintained by the nurses who belonged to the Association for their older fellow members. The freehold property belonged to the Association. In the Home they accommodated nine old nurses. Then the nurses had their own benevolent fund—the Helena Benevolent Fund—for those members who were sick. It was greatly to the credit of the members of the Association that both of those funds were maintained by the working nurses for their less fortunate fellow members. They had a flourishing branch in South Australia, and a very friendly spirit existed between that and the parent Association. Then in regard to the State Registration Bill, previous to 1899 the Association had its own Bill; the Society for the State Registration of Nurses had a Bill; and the Association for the Promotion of State Registration of Nurses in Scotland had a Bill before Parliament. It was pointed out that while those different Bills were before Parliament there was very little chance at all of success for any one of them; so, on the invitation of the Society for the State Registration of Nurses, the different societies of nurses who wished for State registration combined together and drew up an agreed Bill, and the committee which watched over and promoted that Bill was known as the Central Committee for the State Registration of Trained Nurses. That Central Committee represented various influential societies who desired the registration of nurses. The Bill of the Central Committee passed its first reading in the House of Commons. When the war commenced, for patriotic reasons, the Central Committee decided that it would not push forward its Bill; Parliament had quite enough on its hands at the time without having to attend to that controversial measure. So nothing was done about it. The differences between the Bills of the two bodies were three: First of all, the Central Committee said that they wished a Bill which provided for an independent governing council. The general council which should govern the nurses should be quite an independent body, and should have no interests of its own to

**STATE REGISTRATION**—*(continued).*

serve at all. They wished for a General Nursing Council just as the medical men had their General Medical Council, and the midwives had their Central Midwives Board. They did not wish any private interests to interfere with their government at all. They said: "We will set up a General Nursing Council which is quite independent of any private interests whatever." The Bill of the College of Nursing said: "No; we, the College of Nursing, Ltd., will govern the nurses." Another difference was that the College Bill said: "We will have forty-five persons who will govern the nurses"; the Central Committee said: "You may have that number, but the nurses must be represented among those forty-five. The names of the organised societies must be set out in the Bill so that they may elect their own representatives." The College said: "No, you put down the names of those people." The matter in hand that day was not merely one of whether or not they would join this society or that. Let them think out clearly the questions which concerned their individual liberties. It was not a question of deciding whether they wanted State registration or not. She took it that most of them wished to have State registration. The point, however, was which of those two Bills were they going to support. Were they going to place their names on the Register of the Royal British Nurses' Association and promote a Bill for an independent governing body for themselves, which had no interests of its own to serve, and no bias in any direction at all, such a Bill as the doctors and the midwives had—a General Nursing Council in fact; or did they prefer to register with the College of Nursing, Ltd., and push forward the Employers' Bill, which placed the whole control of their profession with that new company? Whichever society they joined, their names would be used to push the respective Bill. She wanted them to make themselves quite clear on all the points of controversy. The Royal British Nurses' Association and the National Union of Trained Nurses had no conflicts of interest on the subject of Registration. Their Association by its Royal Charter had the recognition, so to speak, of the State which said to the public: "This is a body which is eminently worthy to say who are trained nurses and who are not: to say what is for the good of the nursing profession and what is not; to push forward schemes for it; and to state whether this particular nurse is fully qualified or whether she is not." In the eyes of the public it gives a certain status undoubtedly, because they belonged to a body which had been recognised by the State.

MR. HERBERT J. PATERSON, F.R.C.S., then addressed the meeting. He remarked that there was a saying, which he had no doubt was well known to them, that "What Lancashire thinks to-day the rest of England will think to-morrow." In view of their reputation for sound judgment, Miss Macdonald and he thought it well to come to Liverpool to put before them some of the reasons why the amalgamation of the College of Nursing, Ltd., with the Royal British Nurses' Association did not materialise, and to give them some indication of what the future policy of the Association was. Very appropriately in that day's *Times* there appeared a belated reply of Sir Arthur Stanley (Chairman of the Council of the College of Nursing) [this letter will be found in full on page 423] to a letter which he (Mr. Paterson) wrote to the *Times*, and which appeared on January 21. Sir Arthur Stanley was a very clever man; he did not often put anything into writing, and there he (Mr. Paterson) admired his wisdom. In this particular letter, however, Sir Arthur Stanley had given the case away, and had admitted the truth of what he (Mr. Paterson) contended in the letter he wrote to the *Times*. In that letter he contended that the Royal British Nurses' Association were perfectly justified in regarding the alterations made in the Supplemental Charter by the Privy Council as of substantial importance. Sir Arthur Stanley did not admit that, and in his letter to the *Times* that day referred to the clause about which the discussion arose, and went on to say: "He [meaning Mr. Paterson] omits to mention that the next clause (C) of the Supplemental Charter, which was agreed between the Association and the College, about which there is no dispute, and in which the Privy Council made no alteration, expressly provides for the 'institution and conduct of examinations and the grant of diplomas and certificates of proficiency in nursing or any branch of nursing.'" It was quite clear from the context of the Supplemental Charter that that particular clause did not negative the clause before. That was meant to provide for the *amalgamated body, if the amalgamation had taken place, to grant diplomas of proficiency in nursing to those nurses that had already passed* through the period of training and had become fully qualified. The Supplemental Charter expressly provided for specialisation after training, and not before. Sir Arthur Stanley appeared to have thrown that view completely over, and he now said that the alterations in Clause "B" were simply to adapt it to Clause "C," so as to enable the conjoint body to have different curricula and different examinations for all classes of nurses. In other words, Sir Arthur Stanley threw over the one-portal examination, the minimum period of three years' training, and the uniform examination which were given as the policy of the College in February 1916. That was the whole point of his (Mr. Paterson's) contention. The Royal British Nurses' Association stood for one class of nurse only—the nurse who had received a period of three years' training in a general hospital, and had been trained in nursing all classes of disease. As in the medical so in the nursing profession, specialisation must come after qualification and not before it. He thought that was a principle of vital importance; it was one that was recognised by the Fever Nurses' Association, and he believed that he was right in saying that it was recognised by all the organised nurses' societies. When the College was first started, it was clearly the intention of its promoters to introduce the V.A.D. workers within its folds in some way or other, and it was only when that idea was given up that the Royal British Nurses' Association agreed to negotiate as to amalgamation. It was curious that the altered paragraph in the Supplemental Charter re-introduced the very feature to which the Royal British Nurses' Association took such exception at the beginning. It was hardly likely that the words "all classes of nurses" would have been introduced by the Privy Council except at the suggestion of, or at any rate with the cognisance of, one of the parties to the amalgamation. It was certainly curious that that paragraph, which comprised one of the features desired by the College, had been reintroduced, although great exception was taken to it by the Royal British Nurses' Association. He would very much like to know whether the matrons on the College Council approved of the whittling down of the qualifications of the nursing profession. Were they going to admit that different classes of nurses, nurses partially trained, nurses trained in a fever hospital, or in a children's hospital only, and acquainted only with the nursing of those particular diseases, were to be entitled to be placed on a State Register on an equality with fully trained nurses who had had three years' training in all forms of disease in a general hospital? If he might parody a quotation with which he began, he would say: "What the College says to-day it will contradict to-morrow." A paragraph contained a distinct assurance that when the College Bill was passed the nurses on the College Register would be placed on the State Register without payment of any further fee. That was a promise that it was highly improbable the College would ever be able to fulfil. Those nurses *who had paid their guineas relying* on that promise would receive a rude awakening when the Bill for State Registration for Nurses was eventually passed. That, however, did not seem to worry the College, as they regarded their pledges rather lightly. He confessed that he was content to take his place amongst the foolish

**STATE REGISTRATION**—*(continued)*.

nurses, for he regarded that as a pledge. It was an easy matter to make a promise and then when the fulfilment of that promise was claimed to say: "Oh dear, you should not have been such a fool as to think we could have given you such a promise!" He did not like those political subtleties, evasions, and equivocations; he liked to call a spade a spade. Numbers of nurses must have joined the College relying on that promise. The Council of the Royal British Nurses' Association felt that there was no likelihood of that pledge being fulfilled after the alterations in the Charter, and so for their own credit, no less than the credit of the College, they thought they ought not to accept those alterations. What had the Royal British Nurses' Association to offer them? Let him say at once that they promised them that they would not flood the daily press with those objectionable advertisements, backed up by misleading statements. How any self-respecting matron who was supposed to further the interests and to protect the interests of nurses could remain on the College Council in face of that degrading exhibition he could not conceive. He knew how bitterly that charitable appeal was resented by the great body of trained nurses, and if the press refused to give a hearing to the other side they would have to take other means of making their protest public. Those advertisements were degrading to a noble profession. On a recent visit to Manchester Lady Cowdray backed up her appeal with a liberal camouflage of statements, some of which he would like to answer. Lady Cowdray was reported to have said: "Many attempts to introduce Bills into Parliament for the registration of nurses have failed for want of organisation."

### Lay Governors and Hospital Chairmen.

Why, asked Mr. Paterson, had State registration been so long delayed? The reason was not want of organisation. The chief reason was the antagonism of the lay managers of hospitals, supported in some instances, he was sorry to say, by members of the medical profession. Probed to the bottom the question was an economic one. The conditions of labour, the long hours, hard work, unstandardised examinations, and very inadequate remuneration of the nursing profession, were not creditable to this country. Hospitals and nursing institutions were concerned with securing cheap labour, and had largely on that account resisted organisation of nurses in their own defence. But there was a larger issue at stake, and that was the right of laymen to control professional work. It was a curious feature of this country that the expert was always made subservient to the administrator, and the administrator was usually chosen chiefly for his perfect incompetence in regard to the things he had got to administer. If a man were a good bookseller, they put him to manage the Navy; if he were a good grocer, they thought he could manage our food affairs. For the chairman of the large hospital probably a crossing-sweeper was good enough. The medical profession, no less than the nursing profession, had suffered grievously from the cramping effect of unintelligent and ignorant lay control. It was not want of organisation, but the opposition of interested lay persons that had delayed State registration.

Lady Cowdray had apparently not heard of the Trained Nurses' Annuity Fund and the Helena Benevolent Fund. Lady Cowdray had stated that a register of between 8,000 and 10,000 nurses had already been made, and she said that during the course of the second year's working it was hoped to have the whole 70,000 or 80,000 nurses on the College Register. Lady Cowdray was a very sanguine lady. It had taken two years to get 7,000 nurses on the College Register, and when at the Council Meeting last September they asked the Treasurer how many of the 7,000 had paid their guineas, they got no answer. Time would show that the majority of the nurses of this country had not yet sufficiently lost their self-respect as to associate themselves with a body which encouraged that pauperising of the nursing profession. The Royal British Nurses' Association had not been as active during the last twenty years as it ought to have been, but it was going to turn over a new leaf. They believed that the time had now come to make a move forward, and to take a greater part in nursing politics than had been the case in the past. Great as was his admiration for nurses, he had often said (this did not apply to Liverpool) that they had one serious fault—namely, that they were woefully indifferent to their own interests. But he believed that indifference was giving place to interest on the part of the nursing profession. Recent events, at any rate, had stirred up new life in the Royal British Nurses' Association, and the keenness with which many of the nurses had followed the negotiations raised hopes of a brighter to-morrow. The nurses had been thinking and acting for themselves. It was due to the action of their nurse members that the alterations in the Supplemental Charter were refused by the Council. Their President, Her Royal Highness Princess Christian, who for more than thirty years had taken the deepest interest in the welfare of nurses, had firmly maintained all along that the decision on questions such as that should be left to the nurses themselves.

### Not Thrown to the Winds.

The planks of the Royal British Nurses' Association were as follows: First of all State Registration, a one-portal examination, a minimum period of three years' training, and a uniform curriculum. Those were the principles put forward with a flourish of trumpets by Sir Arthur Stanley, in February 1916; in February 1918, in the *Times* of February 8 they were all thrown to the winds. [See the actual letter, p. 424, last paragraph, which proves this to be unfounded.]

There were two Bills for State Registration before them in the field at the present time, as Miss Macdonald had told them. There was the Bill promoted by the College, which was a mere skeleton; it had no flesh, not even clothes, and was not fit to see the light of day. The Bill promoted by the Central Committee was all ready to go to Parliament. In the College Bill the names of the Provisional Council who were to make the rules which the nurses had to obey were not given; there was no mention of a one-portal examination or a minimum period of training, or a uniform curriculum. The Bill promoted by the Central Committee established a General Council for the training and registration of nurses. Amongst the most important duties and powers of that Council were the standardisation of training, and the examination and registration of nurses. The Bill promoted by the Central Committee was supported by the Royal British Nurses' Association, by the British Medical Association, and, he believed he was right in saying, by all the organised nurses' societies in England, and by some of those in Scotland and Ireland. To get that Bill through was to be the endeavour and aim of the Royal British Nurses' Association.

Dr. E. W. Hope (the Chairman) said he had listened attentively both to Miss Macdonald and Mr. Paterson, and without for one moment assenting to many of the propositions put forward by them, he thought their addresses would serve an extremely useful purpose. They had been definite, plain, and clear. In no sense had they minced matters. He hardly, however, concurred with the view that the appeal—of which he had that afternoon seen the advertisement for the first time—could be looked upon as so offensive and distressing as it appeared to have been to the speakers. (Applause.) To his mind, that appeal and the appeals which medical men made on behalf of their brethren who had been less successful were very similar. The College of Nursing, although it was not for him to defend it, did appear to have exercised one useful impulse; and that was, it had stimulated the Royal British Nurses' Association, which

**STATE REGISTRATION—(*continued*).**

had enjoyed this Royal Charter for upwards of thirty years. The latter body, perhaps a little bit weighted down by its Charter, had now at length been galvanised into life, as Mr. Paterson had told them. Personally, he could not help feeling that out of these controversies agreement did eventually come. He was sanguine that a meeting such as that really made for agreement. At all events, they saw perfectly clearly what the sentiments of one side were, if they were not equally well-favoured with the sentiments held in regard to the other society—they perhaps had been a little more diffident. But without asking them for any more definite expression of their views, he could not help feeling that the differences which divided the two bodies were very small, and he looked forward with great hope to see a really strong and united Nursing Service, which would, as he said at the start, result in greater efficiency of nursing, and what he felt was intimately associated with and inseparable from efficiency in nursing—viz. the welfare, and the promotion of the welfare, of the nurses themselves. That was what he looked for. (Applause.)

Other Speakers.

Miss Gibson expressed her absolute astonishment at the attitude which had been displayed that day towards the British Women's Hospital Committee. How anybody could call the appeal a charitable appeal she could not understand. The nation desired to pay a portion of the debt—and such a debt—as it owed to the nurses. There was no question of charity in paying what was due. Nurses, next to the sailors and soldiers, have done more than any other part of the community. Miss Macdonald nad referred to the work of the R.B.N.A. Miss Gibson went with Miss Macdonald to see the Benevolent Home in Clapton Square, London. It contained nine people. That was the complete provision which one large Association had been able to make for the nursing profession. Mr. Paterson had referred to the Trained Nurses' Annuity Fund—which Fund, as they perhaps knew, was founded with part of the money collected from the nation as a reward for Miss Nightingale for all her work in the Crimea. Let them go back to the foundation of trained nurses in this country, and they would find it was laid through the means of a national tribute fund to the lady who instituted the trained nurse by her work in the Crimea. There was no real difference between the Nightingale tribute and the appeal now being made, despite Miss Macdonald's and Mr. Paterson's outbursts. Lady Cowdray and her Committee were simply the means whereby a debt by the nation to a great body of women who had served the nation faithfully and well could be discharged. Miss Gibson added, if the nation and the supporters of the two speakers referred to had come to that state where they did not look upon it as an honourable and straight thing to pay their debts, she was very sorry for the nation No amount of money could pay the debt which the nation owed to the nurses. Referring to the Trained Nurses' Annuity Fund, she asked Miss Macdonald and Mr. Paterson and their friends, when they said they did not require to get help from the nation, were they aware that there were dozens of nurses waiting for years and years to get a pittance from that Fund? They had heard a great deal about lay representation on the College Council. There were on that Council 3 laymen, 10 doctors, and 20 nurse members. She did not quite know where the lay element came in. She was very sorry for these three laymen, because they were not allowed to say much. There had never been any question in the College Council of V.A.D., or untrained nurses, or half-trained nurses, being admitted to the register. None of these things had ever been even thought of. This had been denied and demonstrated as false over and over again. Who gained by this repetition of unfounded libels? Miss Gibson further said she could affirm on her own authority, that thousands of nurses saw the propriety of their getting some recognition from the nation, and thousands of nurses were willing and anxious to join the College. The College of Nursing stood for the State registration of nurses. It had always stood for it, and it was standing for it still. It stood further for a uniform curriculum, a one portal examination, and not less than three years' training in a recognised training-school. No one would be in the College who was not a fully-trained nurse. Miss Gibson declared she had no objection to the appeal advertised in the *Times*, and she denied that she or any other matrons who supported the Nation's Tribute to Nurses were wanting in self-respect.

Dr. MacWilliam maintained that the great point was to try to bring the opposing bodies or sections of the nursing world together. Miss Garnett, who was present when Lady Cowdray spoke, said all Miss Macdonald's questions would be replied to at a subsequent meeting. She pointed out that the great Universities had benefited by public gifts, and for three centuries some of the greatest statesmen and literary men in the country had benefited by the charitable endowments given to the Universities, to the no small benefit of the whole nation. The sum of £250,000 mentioned in the appeal of the British Women's Hospital Committee was less than one-thirtieth of the sum that the nation was now spending daily on the war. Mr. C. A. W. Roberts (Poor Law) said, "Surely to goodness there were able men and able women who could induce them to sink their petty differences for the benefit of the nurses." Dr. Biggs proposed, and Dr. Hayward, who favoured the settlement of all differences in the nursing world, seconded the vote of thanks to the Chair. He further said, referring to the Nation's Tribute and Endowment Funds, he did not see anything to go away ashamed about that such Funds were being raised. They were not charity, they represented the Nation's gratitude. The vote of thanks was duly passed and the meeting terminated.

